# Acute Myelopathy as Initial Presentation of Disseminated Histoplasmosis: A Case Report

**DOI:** 10.1155/crdi/1124517

**Published:** 2025-04-03

**Authors:** Lisle Blackbourn, Elisa Yoo, Michael D. Lunt, Umair Hamid

**Affiliations:** ^1^Department of Neurology, University of Illinois College of Medicine Peoria, Peoria, Illinois, USA; ^2^Neurology, OSF Illinois Neurological Institute, Peoria, Illinois, USA; ^3^Department of Internal Medicine, University of Toronto, Toronto, California, USA; ^4^Department of Pathology, OSF Saint Francis Medical Center, Peoria, Illinois, USA; ^5^Neurological Institute, Cleveland Clinic, Cleveland, Ohio, USA

**Keywords:** acute myelopathy, case report, CNS histoplasmosis, disseminated histoplasmosis, immunocompromised

## Abstract

Histoplasmosis is a fungal infection caused by the fungus *Histoplasma capsulatum* that can rarely present with central nervous system (CNS) manifestations that include meningitis, encephalitis, focal brain or spinal cord lesions, and stroke syndromes. Because of this variation from patient to patient, CNS histoplasmosis is a difficult clinical diagnosis to make, which can be further hindered by no highly sensitive diagnostic testing available. Here, we present a unique case of a 46-year-old male immunocompromised due to type 1 diabetes mellitus with disseminated histoplasmosis as an acute presentation of myelopathy. Patient had left leg weakness for a few days prior to presentation and a neurological exam remarkable for signs of acute thoracic myelopathy, specifically concerning for Brown-Séquard syndrome. MRI imaging demonstrated an enhancing thoracic spinal cord lesion along with multiple cerebral enhancing lesions, bilateral adrenal masses, and innumerable pulmonary nodules. Biopsy results demonstrated yeast forms consistent with Histoplasma species.

## 1. Introduction

Histoplasmosis is a fungal infection caused by the fungus *Histoplasma capsulatum* that can rarely present with central nervous system (CNS) manifestations that include meningitis, encephalitis, focal brain or spinal cord lesions, and stroke syndromes [1]. *Histoplasma capsulatum* is known to be endemic to the Ohio and Mississippi River Valleys in the United States as well as much of Latin America but can be found elsewhere in the world [2, 3]. It has been reported the yearly infection rate is 1%, with an estimated 3 million cases in the US each year based on census data [2].

In disseminated histoplasmosis, it is estimated that 5%–10% of the cases will involve the CNS [1]. There are no single tests for CNS histoplasmosis that have high sensitivity and, therefore, the use of multiple testing, such as cerebrospinal fluid (CSF) culture, antigen testing, and serologic testing, is recommended. Recommended antifungal treatment varies based on presentation and anatomical locations of infection [4].

Myelopathy has been reported as a presentation of known histoplasmosis [5]. There are also reports of indolent histoplasmosis as an isolated intramedullary enhancing spinal cord lesion with no myelopathy concern [6]. If there was reporting of acute myelopathy with histoplasmosis, such patients also had other neurological presentations such as strokes, encephalopathy, and hydrocephalus [7]. Here, we present a case of a 46-year-old male presenting with complaints of left lower extremity weakness, bilateral leg numbness, back pain, personality change, urinary retention, weight loss, and constipation. The uniqueness in our case lies in the initial presentation which was solely an acute myelopathy of disseminated histoplasmosis.

## 2. Case Presentation

A 46-year-old male with a past medical history of type 1 diabetes mellitus, left posterior cerebral artery (PCA) stroke with residual deficits of right homonymous hemianopia and right-sided sensory deficit, past diabetic ketoacidosis episode, hypertriglyceridemia, and coronary artery disease presented to the emergency department for left leg weakness that started 6 days prior. He also reported paresthesias around his lower abdomen that descended down to his lower extremities along with urinary retention and constipation that all started within the 6 days.

Upon further questioning, the patient had first noticed back pain that started 4 months prior to presentation that progressively worsened. One month prior, he started to notice a gradual onset of muscle stiffness, cramping, and numbness in his left foot. The muscle stiffness would resolve after a few minutes initially but gradually progressed to impairment of dorsiflexion. Around the same time, he reported being confused with his finances, which he never had difficulty with prior. He also was having a change in personality including changes in food preference and short temper with his pets. He then noticed he had circumferential numbness around his abdomen at the level of the lesion. About 6 days prior to presentation to the hospital, he was having irregular bowel movements, urinary incontinence, and loss of sensation around his groin. The patient also reported a 12-pound weight loss during this time, despite eating in excess to try to gain weight.

On the initial neurological exam, he had subtle weakness in his left proximal and distal lower extremity with spasticity. He had increased left-sided reflexes in his patella with crossed adductor reflex and ankle along with extensor response of his toe. He also had a loss of vibration and proprioception sensation contralaterally to the weakness along with decreased pinprick and temperature ipsilaterally concerning for Brown-Séquard syndrome.

**Figure 1:**
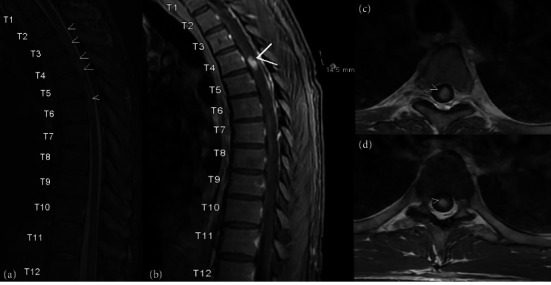
MRI T-spine with and without contrast. (a) T2 sagittal view with hyperintensities at multiple levels from T2–T6. (b) Fat suppressed contrast-enhanced T1 sagittal thoracic spine image with hyperintensity at the T4 level. (c-d) T1 postcontrast axial view of T4-T5 with hyperintensity intramedullary showing contrast enhancement.

Based on a potential acute myelopathy presentation, the patient underwent stat MRI C-, T-, and L-spine with and without contrast imaging. MRI T-spine demonstrated Gadolinium enhancing and mildly T2 hyperintense 0.7 × 0.7 × 1.4 cm intramedullary lesion centered within the central spinal cord at the T4 level as seen in Figure 1. No significant associated cord enlargement was identified.

**Figure 2:**
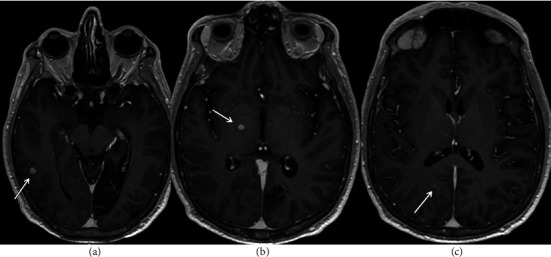
MRI brain with and without contrast showing numerous enhancing lesions.

MRI brain was completed thereafter with numerous enhancing lesions nonspecific in nature but concerning for disseminating encephalomyelitis versus a vasculopathy or coagulopathy with a potential infectious, granulomatous, or inflammatory etiology as seen in Figure 2.

Lumbar puncture was then performed and neurosurgery was consulted on with recommendations for decadron use and awaiting CSF results. Results of the lumbar puncture can be seen in Table 1 and showed no significant results outside of an elevation in CSF glucose.

Flow cytometric analysis on the CSF showed scant cellularity without significant discrete lymphoid or progenitor cell population.

CT chest/abdomen/pelvis was completed, demonstrating bilateral adrenal gland masses, innumerable pulmonary nodules in bilateral upper lobes, cortical wedge-shaped irregularity of the left kidney, splenomegaly, enlarged prostate gland, and bladder wall thickening. Oncology was consulted given these findings and the patient's endorsement of unintentional weight loss. Oncology recommended an IR-guided biopsy of the adrenal mass and continuation of steroids. A whole-body PET scan was originally planned but canceled due to issues with hyperglycemia while inpatient.

**Figure 3:**
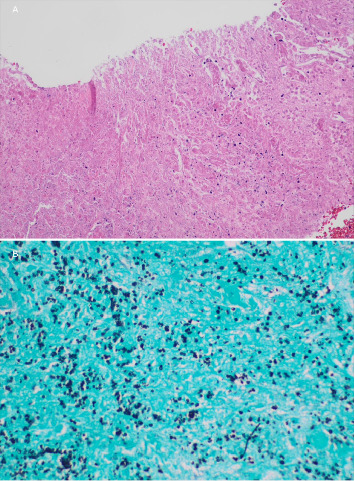
Histology pictures of (A) a hematoxylin and eosin (H&E) stain taken at 200x magnification showing a necrotizing granuloma and (B) a Grocott's methenamine silver (GMS) stain taken at 400x magnification in the same area with small black ovals that represent histoplasma.

The patient then underwent a CT-guided left adrenal mass biopsy. Pathology returned without evidence of cancerous properties and thus planning for radiation was canceled. Evidence of corroborated yeast cells found on histology staining of the left-sided adrenal mass biopsy warranted further infectious workup, demonstrating necrotizing granulomatous inflammation consistent with Histoplasma as seen in Figure 3.

Infectious diseases were consulted and recommended IV amphotericin B for 14 days, steroid discontinuation, and further testing for Histoplasma of the CSF sample. Infectious diseases added Histoplasma antigen, quantitative enzyme immunoassay random urine for which resulted positive. T and B cell testing was added to the CSF sample with negative results. After only 2 days of treatment, the patient endorsed significant improvement in sensation and showed increased strength of the left lower extremity. By the date of discharge, the patient had a normal neurological exam outside of his chronic stroke deficits. Following the 14 days of IV amphotericin B, the patient was placed on itraconazole for 1 year. Repeat MRI brain and T-spine imaging was done 2 weeks postamphotericin B therapy and showed interval decreases in both the spinal lesion and the multiple brain lesions. Urine Histoplasma antigen also later tested negative months after treatment initiation.

It was later found out that a year prior to his presentation, the patient went to an old barn in Pontiac, Illinois, that was covered in pigeon and bat excrement. This may have been an event of fungal exposure. The patient also traveled to Pocahontas, Arkansas, in October 2022, another endemic area for histoplasmosis.

## 3. Discussion

Our patient had yeast forms on biopsy that were consistent with Histoplasma species along with a positive urine Histoplasma antigen. It should be noted that urine Histoplasma antigen can have cross-reactivity with other fungal species such as Blastomyces. However, treatment is the same for these two fungi and, therefore, differentiation is usually not required. In our case, it was determined to be Histoplasma as the biopsy yeast forms were consistent with Histoplasma. Histoplasma has budding yeast that is connected at a narrow base, which helps distinguish it from that of Blastomyces [2]. Shape, size differences, and wall thickness also help distinguish between various fungi on staining.

The diagnosis of disseminated histoplasmosis can be challenging as differential diagnoses, such as metastatic cancers or abscesses, are more prevalent. CNS histoplasmosis requires a higher degree of clinical suspicion [2]. The diagnosis of histoplasmosis was challenging in our patient. He was initially set up to receive radiation therapy by oncology as the differential of suspected metastatic carcinoma was highest with other symptoms such as weight loss, disseminated lesions in the brain, and a history of smoking. Fortunately, radiation was not initiated and the patient returned from the radiation suite the moment pathology came back positive for histoplasmosis. Given the patient's unintentional weight loss of 14 pounds, numerous brain-enhancing lesions, an enhancing intramedullary spinal cord lesion, and CT chest/abdomen/pelvis showing bilateral adrenal masses, innumerable tiny pulmonary nodules in the bilateral upper lobes, and splenomegaly, there was reasonable concern for metastatic carcinoma.

This is not an uncommon scenario. One retrospective study showed that only 35% of the patients were correctly diagnosed with histoplasmosis when initial CNS symptoms had been present for 2 weeks or less [8]. A total of 12% of patients in this study were correctly diagnosed after having CNS symptoms for more than 26 weeks, highlighting the difficulty in making a diagnosis of histoplasmosis. This rate increased to 27% when considering any symptom of histoplasmosis infection that had been present for more than 26 weeks. Only 28% of the patients within 2 weeks of presentation of any symptoms were correctly diagnosed. One reason disseminated histoplasmosis and CNS histoplasmosis may be overlooked is that it is generally associated with immunocompromised individuals. This retrospective study also surprisingly had determined that 29% of the patients were not immunosuppressed.

Such diffuse CNS involvement with disseminated histoplasmosis has rarely been reported and rare reports of intramedullary spinal cord histoplasmosis [5, 9, 10]. A literature review done in 2023 for intramedullary spinal cord histoplasmosis found that 50%–90% of immunocompetent individuals with histoplasmosis were asymptomatic [9]. About 1/3rd of cases found in this review were determined to be immunocompetent individuals while 2/3 rds were observed in immunocompromised individuals. Immunocompromisation also increases the risk of disseminated histoplasmosis by upto 10 times [9].

The current Infectious Diseases Society of America guideline revised in 2007 for CNS histoplasmosis is liposomal amphotericin B 5.0 mg/kg daily for a total of 175 mg/kg given over 4–6 weeks followed by itraconazole 200 mg either 2 or 3 times daily for at least 1 year [4]. It is also recommended that itraconazole be continued until resolution of any CSF abnormalities, which also include Histoplasma antigen CSF levels.

## 4. Conclusion

CNS histoplasmosis is a rare presentation of disseminated histoplasmosis that can greatly vary in presentation. Because of this variation from patient to patient, CNS histoplasmosis is a difficult clinical diagnosis to make, which can be further hindered by no highly sensitive diagnostic testing available. Further research into a highly sensitive rapid diagnostic test would help aid in the diagnosis of histoplasmosis.

## Figures and Tables

**Table 1:** CSF results of lumbar puncture performed on the patient.

Test (parameter)	Result
Culture growth	No growth
Glucose level	104 (normal range: 40–70 mg/dL)
Protein	41 (normal range: 12–60 mg/dL)
Cell count/TNC	2
MOG CSF testing	Negative
NMO CSF testing	Negative
Paraneoplastic panel	Negative
ACE level	Normal
Enterovirus	Negative
Herpes Simplex 1/2	Negative
Cytology	Negative for malignancy
Fungus or yeast culture	No growth
Histoplasma antigen	No evidence

## Data Availability

All data generated or analyzed during this study are included within the article.
